# Proteomic Strategies on the Management of Phytopathogenic Fungi

**DOI:** 10.3390/jof11040306

**Published:** 2025-04-11

**Authors:** Aldrey Nathália Ribeiro Corrêa, Ana Carolina Ritter, Adriano Brandelli

**Affiliations:** 1 Laboratory of Nanobiotechnology and Applied Microbiology, Institute of Food Science and Technology, Federal University of Rio Grande do Sul, Porto Alegre 91501-970, Brazil; aldreyr@outlook.com (A.N.R.C.); anacarolina.ritter@gmail.com (A.C.R.); 2 Center of Nanoscience and Nanotechnology, Federal University of Rio Grande do Sul, Porto Alegre 91501-970, Brazil

**Keywords:** antifungal agent, fungal proteomics, mass spectrometry, plant disease, sustainable agriculture

## Abstract

Phytopathogenic fungi are important causative agents of many plant diseases, resulting in substantial economic losses in agriculture. Proteomics has become one of the most relevant high-throughput technologies, and current advances in proteomic methodologies have been helpful in obtaining massive biological information about several organisms. This review outlines recent advances in mass spectrometry-based proteomics applied to the study of phytopathogenic fungi, including analytical platforms such as LC-MS/MS and MALDI-TOF, as well as quantitative strategies including TMT, iTRAQ, and label-free quantification. Key findings are presented from studies exploring infection-related protein expression, virulence-associated factors, post-translational modifications, and fungal adaptation to chemical fungicides, antimicrobial peptides, and biological control agents. Proteomic analyses have also elucidated mechanisms of resistance, oxidative stress response, and metabolic disruption following exposure to natural products, including essential oils and volatile organic compounds. The proteomic approach enables a comprehensive understanding of fungal biology by identifying proteins related to pathogenicity, stress adaptation, and antifungal resistance, while also facilitating the discovery of molecular targets and natural compounds for the development of sustainable antifungal strategies that reduce risks to human health and the environment.

## 1. Introduction

Phytopathogenic fungi are among the most challenging threats to global agriculture, causing significant crop yield and quality losses. These fungi possess highly adaptive mechanisms, enabling them to survive under diverse environmental conditions and resist traditional control measures [1]. The need for innovative strategies to manage these pathogens has driven a surge in proteomics-based research, which offers a molecular-level understanding of fungal responses to antifungal agents and their mechanisms of pathogenicity [2,3].

Proteomics has become a valuable and powerful tool for investigating the molecular complexities of fungal pathogenicity. By examining protein expression, modifications, and interactions through proteomic studies, it is possible to identify critical virulence factors and stress response mechanisms. For instance, proteomic analyses of *Botrytis cinerea* have elucidated the role of its surfactome in host–pathogen interactions, highlighting essential proteins for fungal virulence and survival [4]. Similarly, proteomic studies on *Cochliobolus heterostrophus* have shown that isothiocyanates (ITCs) significantly disrupt energy metabolism and cell wall integrity, thereby impairing fungal growth and pathogenicity [5].

A fundamental aspect of successful proteomic studies is the preparation of high-quality samples. Effective sample preparation ensures the integrity and representativeness of extracted proteins, which is crucial for accurate downstream analysis. Advanced techniques such as iTRAQ and LC-MS/MS have further improved the resolution and sensitivity of proteomic studies, allowing researchers to identify differentially expressed proteins and understand their roles in fungal physiology [6,7]. For example, the proteomic response of *Colletotrichum gloeosporioides* to volatile organic compounds (VOCs) from *Bacillus subtilis* revealed significant alterations in cell wall biosynthesis and energy metabolism, demonstrating the potential of VOCs as biocontrol agents [8]. These methodologies were further refined and used to study complex interactions in fungal biology, such as stress responses mediated by MAP kinase pathways in *Magnaporthe oryzae* [1].

Recent advances in proteomics have diversified the methodologies available to study fungal biology and antimicrobial resistance. Techniques, such as MALDI-TOF MS, have enabled the modernization of diagnostic microbiology, allowing the rapid identification of fungi and their susceptibility profiles to antifungal agents, being particularly critical for combating multidrug-resistant pathogens [9]. By analyzing proteomic profiles of fungal isolates exposed to antifungal drugs, MALDI-TOF MS has been used to detect drug-induced proteome changes, revealing resistance mechanisms and informing treatment strategies [9,10]. Moreover, the proteomic exploration of filamentous fungi, including their secretomes, has unveiled enzymes and proteins crucial for lignocellulose degradation and biotechnological applications. Challenges in sample preparation, such as protein extraction from robust fungal cell walls, have led to the development of refined protocols that maximize protein yield while minimizing contamination and degradation [10].

The development of novel antifungal agents is another critical area where proteomics plays a pivotal role. As resistance to conventional fungicides becomes increasingly prevalent, there is an urgent need for alternative strategies to combat fungal pathogens. Proteomics has been instrumental in identifying molecular targets and resistance mechanisms, as demonstrated by studies on *M. oryzae* revealing that overexpression of specific phosphatase genes confers resistance to fludioxonil, a widely used fungicide [3]. Similarly, the proteomic approach showed that the inhibitory effects of microbial secondary metabolites, such as benzothiazole, on *B. cinerea* can be attributed to disruptions in mitochondrial function and energy production pathways [6].

Recent developments in proteomics have also enabled a deeper exploration of host–pathogen interactions. Studies on *Ustilago maydis* have provided insights into the role of effector proteins in modulating host immune responses and promoting fungal virulence [2]. These findings not only improve our understanding of fungal biology but also pave the way for innovative approaches to disease management. Moreover, proteomics has facilitated the discovery of eco-friendly antifungal alternatives. Studies on VOCs from *Saccharomyces cerevisiae* and *Bacillus subtilis* have demonstrated their ability to inhibit fungal growth by targeting metabolic and cellular processes critical for survival [8,11].

The integration of proteomics with transcriptomics and metabolomics offers a holistic view of fungal responses to antifungal agents. This multidisciplinary approach has been exemplified in studies on *Phyllosticta citricarpa*, where proteomic analysis revealed that VOCs interfere with key metabolic pathways, while transcriptomic data provided insights into gene regulation under antifungal stress [8,11]. These integrated studies highlight the potential of proteomics to fill knowledge gaps and drive the development of effective antifungal strategies [11,12].

In this context, proteomics has emerged as a transformative tool in the study of phytopathogenic fungi. By uncovering the molecular basis of fungal responses to antifungal agents, proteomics not only enhances our understanding of fungal biology but also informs the development of innovative solutions for crop protection [1]. Although the study of fungal pathogens through proteomics approaches has been described in some review articles, the focus is devoted to fungi of medical importance [13,14,15]. Therefore, the current scientific literature presents a gap regarding systematic reviews on the application of proteomic analyses to understand the biological mechanisms of phytopathogenic fungi. The application of proteomics in agriculture is still under development, with a limited number of studies addressing the various proteomic strategies available [9,16,17]. This review delves into key proteomic studies, highlighting their contributions to addressing the challenges posed by phytopathogenic fungi and their implications for sustainable agriculture.

## 2. Bibliography Searching

**Figure 1:**
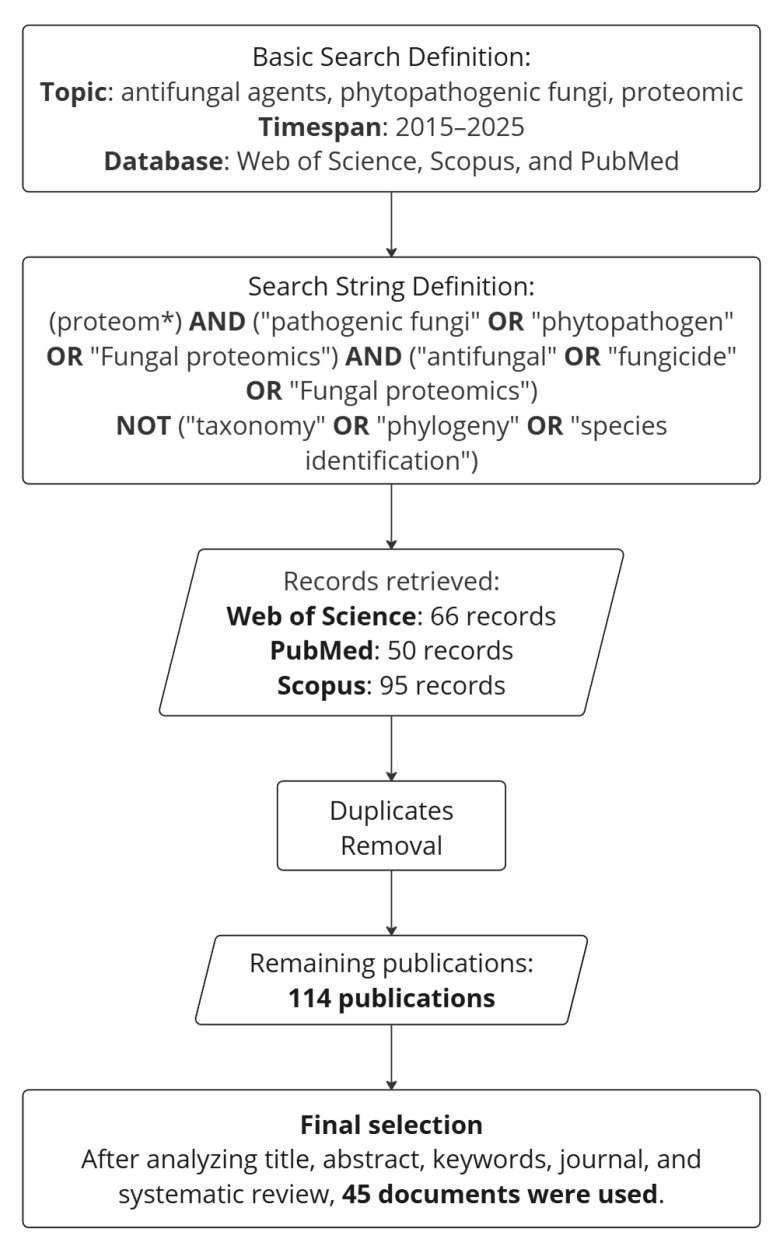
Flow diagram of the searching strategy used to prepare this work. The asterisk (*) is a Boolean wildcard used to retrieve terms with the same root (e.g., ‘proteom’).

A bibliometric analysis was performed to search the most relevant articles, considering the novelty of proteomic methods that have been helpful in understanding the mechanisms of antifungal agents in phytopathogenic fungi. The first step was to define the keywords, databases used to search for studies, Boolean operators, and relevant filters (Figure 1). The selected search strings were submitted to the following databases: Web of Science, Scopus, and PubMed. After the first run, duplicates were removed, and a systematic review of titles, abstracts, keywords, and full texts was performed, resulting in the selection of 45 publications.

**Figure 2:**
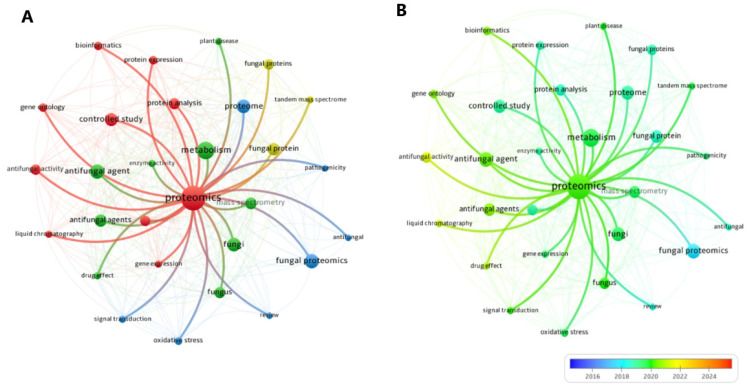
(**A**) Network visualization of the co-occurrence map of terms related to “Proteomics”. The nodes represent key scientific terms extracted from publications, and the connections indicate the frequency of co-occurrence between these terms. The size of the nodes reflects the relevance or frequency of the terms within the research context. Distinct colors represent thematic clusters, such as antifungal agents (green), metabolism (yellow), fungal proteomics (blue), and protein analysis (red). Thicker lines indicate stronger associations between the terms, highlighting key research relationships within the field of proteomics. (**B**) Overlay visualization of the co-occurrence map of terms related to “Proteomics”. The central node represents ’Proteomics’, with connected nodes indicating related research topics. Node size reflects the frequency of occurrence, while edge thickness represents the strength of associations between terms. The color gradient (blue to yellow) indicates the average publication year, with blue representing older topics (2016–2018), green representing intermediate topics (2019–2021), and yellow highlighting emerging topics (2022–2024).

The network analysis maps were made by VOSviewer software version 1.6.20 (https://www.vosviewer.com/). The minimum number of relationships with terms in the use of VOSviewer was set to 10 terms. The visualization was presented as the co-occurrence maps of terms related to “Proteomics” (Figure 2).

## 3. Proteomics as a New-Generation Tool for Studying Antifungal Agents

In the last decade, an increasing number of proteomics approaches have been developed, particularly to uncover the key mechanisms of action of antifungal and antibacterial agents [18]. Compared to other omics approaches, such as transcriptomics, proteomics offers diverse advantages, including the ability to directly analyze functional molecules involved in specific biological processes [4].

Phytopathogenic fungi are a critical group of microorganisms responsible for several problems in agriculture worldwide. The most common phytopathogenic fungi, *B. cinerea*, *Fusarium* spp., *Phythophora* spp., and *Alternaria alternata*, are responsible for infecting many crops and fruits [1,6,19,20]. Genetic engineering represents an additional strategy to control pathogens, complementing traditional approaches such as biological control, chemical treatments, and the breeding of resistant varieties. Chemical control is performed with synthetic fungicides, and although it is an efficient way to eliminate phytopathogens, it can result in chemical residues, development of resistance, environmental contamination, and potential health risks [20,21]. Therefore, the biological control of fungal diseases has been employed as a green prevention strategy, ensuring safety for humans and non-target organisms while reducing the development of microbial resistance.

**Figure 3:**
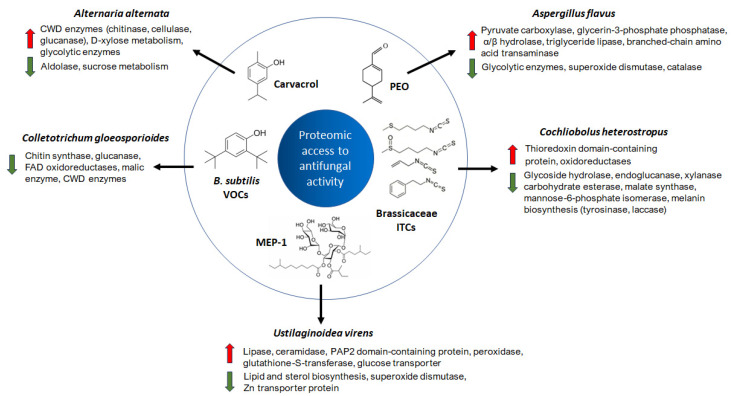
Proteomic access to antifungal mechanisms of natural compounds. Red and green arrows indicate upregulated and downregulated DEPs, respectively. CWD cell wall degrading enzymes; PEO, perilla essential oil; VOCs, volatile organic compounds; ITCs, isothiocyanates; MEP-1, trisaccharide ester from *Pezicula neosporulosa*.

Proteomics tools have been used to study the antifungal mechanisms of various natural compounds (Figure 3). Carvacrol is a plant-derived terpenoid presenting efficacy to control *Alternaria alternata*, a pathogen responsible for rot in various fruits and vegetables [20]. Carvacrol treatment induces changes in the content of several carbohydrates in *A. alternata*, and transcriptomic and proteomic analyses revealed accelerated decomposition of cell wall polysaccharides in the fungi besides hindered sucrose consumption and promoted the decomposition of carbohydrates related to stress resistance. In summary, carvacrol affects the growth, infection, and stress resistance of *A. alternata* [20]. The potential antifungal properties of the essential oil extracted from *Perilla frutescens* (PEO) were investigated using the LC-MS/MS technique. The study identified significant alterations in polysaccharides, proteins, and lipids in *Aspergillus flavus* following treatment with PEO. The findings indicated that PEO disrupted energy metabolism and defense mechanisms, leading to impaired fungal growth. Proteomic analysis revealed that the antifungal effect of PEO was primarily linked to the inhibition of antioxidative defense systems and the glycolysis pathway. In response to the imbalance in glycolysis, *A. flavus* activated alternative energy-producing pathways to compensate for the disruption [12]. These findings highlight the potential of PEO as a natural antifungal agent, offering an alternative approach to controlling *A. flavus* and preventing aflatoxin contamination in cereals.

*Bacillus* species, such as *B. subtilis* and *B. velezensis*, are commonly used as biological control agents against plant pathogens due to their production of varied antimicrobial compounds. The inhibitory effects of VOCs produced by *B. subtilis* CF-3 on *C. gloeosporioides* were evaluated by liquid chromatography tandem mass spectrometry (LC-MS/MS) and concluded that *B. subtilis* CF-3 has potential application in postharvest protection of fruits and vegetables [8]. In another study, the global proteomic response of *B. velezensis* to fungal plant pathogens was investigated using mass spectrometry [7]. The authors observed that all tested strains produced lipopeptides, such as surfactin and bacillomycin, and that proteomic analysis offered a more detailed understanding of the adaptation and response mechanisms of fungi–bacteria interaction, including an increased abundance of proteins involved in the biosynthesis of antimicrobial compounds.

The secretome of five *Lactiplantibacillus plantarum* strains showing inhibitory activity against bacteria and fungi was analyzed using a shotgun proteomic approach and label-free quantitative analyses [22]. The results showed that 602 proteins were present in the secretome of the analyzed strains, some of which were chitin-binding proteins. Chitin is an essential component of the fungal cell wall, so chitin-binding proteins are important in controlling fungal growth.

Isothiocyanates (ITCs) are secondary metabolites produced by Brassicaceae, which have significant antagonistic activity against pathogenic fungi, including *Cochliobolus heterostrophus* [5]. The proteomic analyses helped to elucidate the underlying antagonistic mechanism of ITCs on fungal pathogens, as the inhibition of *C. heterostrophus* by A-ITC downregulated the expression of genes related to energy metabolism, oxidoreductase activity, melanin biosynthesis, and cell wall degrading enzymes.

The need for the development of safe and effective antifungal agents to control *Ustilaginoidea virens*, an ascomycetous fungus that causes rice false smut, a devastating emerging disease worldwide, has been highlighted [23]. A novel trisaccharide ester from the metabolites of *Pezicula neosporulosa* SC1337, and it could be observed through proteomic and transcriptome experiments that MTE-1 inhibited the growth of *U. virens* by preventing lipid synthesis and altering primary metabolic pathways, allowing for the future application of MTE-1-derived agents in the treatment of fungal diseases [24].

As mentioned above, in phytopathogenic fungi, proteomic approaches have emerged as an essential tool for advancing our understanding of the molecular mechanisms underlying the efficacy of biological antifungal agents. By providing a comprehensive view of protein expression, interactions, and post-translational modifications, proteomics allows the identification of key molecular targets and pathways affected by these agents.

## 4. Proteomic Approaches in Fungal Virulence and Pathogenicity

Phytopathogenic fungi, especially Ascomycetes and Basidiomycetes, are a major cause threat to global agriculture yield loss in large-scale agricultural production [25]. These microorganisms show a very high versatility during their infection cycles, employing highly specific and unique mechanisms to infect and colonize host plants. Therefore, understanding the key factors that influence virulence, including effector proteins, enzymes that degrade host cell walls, and molecules that suppress plant immune responses, is critical for developing innovative strategies to mitigate plant diseases.

Virulence and pathogenicity analyses in fungi can be performed using various proteomic methods. Differentially expressed proteins were characterized among *Rhizoctonia solani* isolates using three distinct proteomic approaches [26]. Initially, the protein profiles of 20 *R. solani* isolates with varying disease severity were analyzed by SDS-PAGE. This technique revealed that protein bands, particularly those between 25 and 180 kDa, were common to most isolates, while lower molecular weight bands showed greater variation. In addition, 2-DE and LC-MS/MS were used as proteomic strategies to compare the mycelial proteins of highly virulent and less virulent isolates. The results showed that proteins known to play critical roles in virulence and pathogenicity, such as D-arabitol dehydrogenase 1 (carbohydrate metabolism), deoxyhypusine synthase (protein modification), and 1,4-β-glucanase (plant cell wall degradation) [26].

Glycosylation is one of the most common post-translational modifications (PTMs) of proteins, and it is involved in many molecular and cellular functions. Knowing this importance and the paucity of studies on this topic, a quantitative glycoproteomic study was conducted to elucidate the effects of antifungal volatiles (AVs) on *F. graminearum* using hydrophilic interaction liquid chromatography (HILIC) and high-resolution mass spectrometry [27]. The study revealed that fungicide treatment significantly down-regulated glycosylation, particularly in the cell wall, membrane, and extracellular regions, affecting fungal metabolism, protein synthesis, glycosylation processes, and protease activity. These changes suggest that a mixture of AVs produced by marine actinomycete *Streptomyces* sp. CGMCC 4.7292 disrupts fungal development by interfering with key cellular functions.

A quantitative LC-MS/MS-based proteomic approach was used to compare highly and less virulent isolates of *R. solani* and identified 48 differentially expressed proteins, 27 of which were upregulated in the more virulent isolate [28]. Proteins such as sorting nexin four may be involved in intracellular protein trafficking, and squalene synthase is essential for sterol biosynthesis in the fungal cell membrane. Other proteins, including pyranose 2-oxidase, Hsp70, cyanate hydratase, topoisomerase 1, and histone demethylation proteins containing the JmjC domain, were also found to be upregulated in the highly virulent isolate. These proteins are associated with stress response, detoxification, transcriptional regulation, and DNA metabolism, processes that are critical during host infection. These upregulated proteins, together with pyranose 2-oxidase, Hsp70, and cyanate hydratase, may play an important role in the pathogenicity and virulence of *R. solani*, suggesting that these proteins could be potential targets for disease control strategies.

Concerning commercial fungicides, novel antifungal targets in *Taphrina deformans*, a major plant pathogen, were identified using subtractive proteomics, high-throughput virtual screening, and molecular dynamics approaches [29]. Computational proteomics approaches were used to analyze 4659 proteins from *T. deformans*, identifying 189 essential proteins, among which glutamate–cysteine ligase (GCL) stood out for its critical role in glutathione biosynthesis, a key process for fungal survival and pathogenicity. Virtual screening and molecular dynamics simulations revealed strong binding affinities of polyoxin D, fluoxastrobin, trifloxystrobin, and azoxystrobin to the active site of GCL, suggesting its potential as an effective antifungal target.

The phytopathogenic fungus *U. maydis* is a biotrophic, dimorphic plant pathogen that infects maize (*Zea mays*). This fungus is a model organism for plant pathogens because it is well-differentiated and amenable to reverse genetics [17]. Thus, the surface-exposed protein complex in *U. maydis* was investigated because of its essential role in virulence [2]. During maize colonization, *U. maydis* secretes effectors that suppress plant immunity and reprogram host cells. The authors identified a protein complex composed of seven proteins (Stp1–Stp6 and Pep1), all co-regulated and expressed exclusively during infection. Co-immunoprecipitation and mass spectrometry experiments confirmed interactions among these proteins, suggesting that the complex plays a central role in translocating effectors into the host. The findings indicate that this protein complex is crucial for the biotrophic lifestyle of *U. maydis* and represents a promising target for antifungal strategies.

*Penicillium expansum*, a necrotrophic plant pathogen, is responsible for postharvest fruit decay, as well as the production of mycotoxin patulin. This toxic secondary metabolite can cause acute, subacute, and chronic toxicity, including genotoxicity, immunotoxicity, and neurotoxicity. The effect of the antifungal protein PgAFP on the proteome and patulin biosynthesis of *P. expansum* was evaluated [30]. The results of the study demonstrated that proteins related to virulence/pathogenicity were reported in the proteome of *P. expansum* after the PgAFP treatment, including alpha-amylase/endo-polygalacturonase (cell wall degrading enzymes) and glucose–methanol–choline oxidoreductase (energy metabolism), which were found in higher relative abundance. In addition, proteomic analysis revealed that PgAFP treatment induced the overexpression of several proteins directly involved in patulin biosynthesis, such as PatE, PatF, PatN, PatD, and PatB, with fold changes ranging from 2.88 to 9.80.

As previously described in this article, *B. cinerea* is a crucial phytopathogenic fungus, one of the most widespread and destructive disease-causing plant pathogens. Many studies have reported control strategies for this microorganism and its mechanisms of infection. The first proteomic analysis conducted during the germination of *B. cinerea* conidia revealed the importance of proteomic approaches in the search for virulence factors in plant pathogenic fungi [31]. Several proteins associated with virulence and pathogenicity were identified, such as peptidyl-prolyl *cis*-*trans* isomerases, superoxide dismutases, and peroxiredoxins were found to be highly accumulated in ungerminated conidia, suggesting their role in early fungal development and host infection. In addition, enzymes such as mannitol 1-phosphate dehydrogenase and 6,7-dimethyl-8-ribityllumazine synthase were identified as potential virulence factors due to their involvement in stress resistance and energy metabolism.

Another study analyzed the same fungus and carried out the first description of the surfactome of a filamentous fungus [4]. The authors identified different infection stages of *B. cinerea* by LC-MS/MS and identified the proteins exposed on the cell surface during a constitutive stage using glucose as the sole carbon source. The study identified several virulence and pathogenicity-related proteins in *B. cinerea*, particularly those associated with signal transduction, host interaction, and metabolic adaptation, such as peptidyl-prolyl *cis*-*trans* isomerases, scytalone dehydratase (*arp1* gene), mannitol-1-phosphate dehydrogenase and 6,7-dimethyl-8-ribityllumazine synthase.

*Fusarium oxysporum*, due to its significant impact on plant and animal diseases, has also been the focus of proteomic studies [32]. *F. oxysporum* f.sp. *lycopersici* (Fol) is one of the most devastating plant pathogens, exhibiting a complex infection process. Post-translational modifications (PTMs) play crucial roles in various biological processes, with lysine acetylation (Kac) being one of the most widespread PTMs. This modification is regulated by histone acetyltransferases, which add acetyl groups, and deacetylases, which remove them. Given that acetylation/deacetylation is closely linked to the virulence of plant pathogens and that non-histone proteins are frequently acetylated, the authors investigated the role of FolSir2, a cytosolic deacetylase, in *F. oxysporum* f.sp. *lycopersici* virulence using LC-MS/MS [32]. The study identified that *FolSir2* deletion impaired virulence without affecting fungal growth, suggesting that cytoplasmic lysine deacetylation is a crucial regulatory mechanism in plant-pathogenic fungi. Additionally, this mechanism was conserved in *B. cinerea*, indicating its potential as a target for broad-spectrum antifungal strategies.

Furthermore, pathogenicity and virulence mechanisms of the *F. oxysporum* subspecies *cucumerinum* (FOC) were investigated using proteomic analysis comparing highly pathogenic (hp-FOC) and weakly pathogenic (wp-FOC) isolates [18]. The authors identified several proteins involved in pathogenicity, such as peroxidase (FoHPMP-24) and catalase-peroxidase (FoHPMP-30), which convert hydrogen peroxide (H_2_O_2_) into water and oxygen, and glyceraldehyde-3-phosphate dehydrogenase (FoHPMP-35), which helps the fungus attach to the extracellular matrix of host cells and has been implicated by some authors as a virulence factor.

## 5. Proteomic Insights into Fungal Responses to Biological Agents

Biocontrol agents, including bacteria, fungi, and plants, produce a diverse array of antifungal compounds that can suppress the growth of phytopathogenic fungi [11,33]. These natural interactions are a critical component of disease control strategies and are increasingly studied using proteomic tools to elucidate how fungal pathogens respond at the molecular level [21,34]. Analysis of the fungal proteome provides a detailed understanding of the mechanisms by which antifungal agents exert their effects, the adaptive strategies that fungi employ, and the pathways that can lead to resistance [34,35].

A relevant example includes the nonribosomal peptides nunamycin and nunapeptin, produced by *Pseudomonas fluorescens* In5. These compounds exhibit selective antifungal activities, with nunamycin targeting *R. solani* and nunapeptin acting against *Pythium aphanidermatum*. Proteomic analyses utilizing MALDI-TOF imaging mass spectrometry have demonstrated that these peptides interfere with distinct fungal pathways, disrupting vital cellular processes. Interestingly, microbial interactions in the environment can alter peptide synthesis, as seen with the suppression of nunamycin production in the presence of *P. aphanidermatum*. This interplay shows the complexity of biocontrol mechanisms and underscores the value of proteomic tools in deciphering these dynamic interactions [33].

The antifungal compound benzothiazole, a VOC, effectively inhibits *B. cinerea* by targeting mitochondrial function. Quantitative proteomic analyses using isobaric tags for relative and absolute quantitation (iTRAQ) method revealed extensive alterations in mitochondrial processes, including disrupted membrane organization and downregulation of glyoxylate cycle enzymes. Additionally, pathway enrichment analyses highlighted a significant reduction in ATP generation and oxidative phosphorylation pathways. Bioinformatic integration further identified key regulatory proteins affected by benzothiazole, suggesting its role in undermining energy homeostasis and nutrient acquisition by *B. cinerea* [6].

VOCs from *Saccharomyces cerevisiae* CR-1, including various alcohols and esters, have been shown to inhibit *Phyllosticta citricarpa*, a citrus pathogen. Proteomic profiling through 2D-PAGE and LC-MS/MS revealed significant downregulation of glycolytic enzymes and proteins involved in the tricarboxylic acid cycle, suggesting a disruption in energy metabolism. Additionally, proteins involved in oxidative stress responses were reduced, indicating a weakened ability of the pathogen to counteract oxidative damage [11]. Expanding this concept, VOCs produced by *Bacillus subtilis* CF-3 significantly altered the proteomic profile of *C. gloeosporioides*. Tandem mass tag (TMT)-based quantitative proteomics identified the downregulation of proteins related to cell wall integrity, energy metabolism, and ergosterol biosynthesis, further emphasizing the potential of VOCs as eco-friendly antifungal agents by targeting critical metabolic pathways [8].

From another perspective, lipopeptides produced by *Bacillus velezensis* strains, such as surfactin, bacillomycin, and fengycin, induce stress responses in target fungi like *Diaporthe* spp. and *F. oxysporum*. Proteomic studies employing nano-LC-MS/MS revealed that *Diaporthe* spp. responds to lipopeptides by upregulating stress-related proteins and reducing amino acid transport and protein synthesis [7]. The study also highlighted changes in lipid metabolism as fungi attempt to remodel their membranes to counteract lipopeptide-induced stress. Extending the understanding of *Bacillus* species in fungal biocontrol, another proteomic study demonstrated that fengycins from *B. subtilis* BS155 effectively target *Magnaporthe grisea* by inducing extensive oxidative stress and chromatin condensation [36]. Comparative proteomics identified significant downregulation of reactive oxygen species (ROS)-scavenging enzymes, such as superoxide dismutase and catalase, leading to ROS accumulation and mitochondrial membrane potential collapse. Additionally, upregulation of DNA repair proteins and cleavage of poly(ADP-ribose) polymerase (PARP) were observed, indicative of apoptotic-like cell death. Therefore, advanced proteomic techniques, including tandem mass spectrometry and fluorescence microscopy, elucidate the multifaceted mechanisms by which lipopeptides disrupt fungal physiology.

In addition to peptides of microbial origin, plant-derived peptides are currently being designed as antifungal alternatives, and proteomic techniques have been used to determine their cellular targets. The value of advanced proteomic techniques in elucidating the molecular mechanisms of defensin-related peptides can be highlighted, paving the way for novel antifungal strategies to control phytopathogenic fungi. In this regard, antifungal peptides derived from the α-core and γ-core regions of a defensin protein (DefSm2-D) from flowers have been investigated [19]. Employing electrospray ionization mass spectrometry (ESI-MS) and circular dichroism (CD) spectroscopy, the study characterized four synthetic peptides designed to target *Fusarium graminearum*. These peptides exhibited significant antifungal activity, with SmAPα1-21 and SmAPγ27-44 demonstrating the ability to permeabilize fungal membranes and disrupt cellular integrity, as observed by transmission electron microscopy (TEM). The α-core-derived peptides also induced morphological alterations in the fungal cell wall, a unique and promising target absent in plant and mammalian cells.

The iTRAQ-based proteomics approach was used to investigate the response of *F. oxysporum* to antifungal peptides, revealing significant disruptions in mitochondrial processes and energy metabolism pathways [37]. Similarly, label-free quantitative proteomics was employed to study the impact of antifungal protein PgAFP on *P. expansum* [30]. The analysis identified changes in the relative abundance of 237 proteins, with stress-related proteins, including glutathione peroxidase and heat shock proteins, significantly upregulated in response to PgAFP. Furthermore, proteins involved in patulin biosynthesis, such as PatE and PatF, were highly expressed, leading to a marked increase in patulin production. These findings highlight how proteomic strategies reveal intricate molecular responses in plant pathogenic fungi, emphasizing disruptions in energy pathways and oxidative stress as essential mechanisms underpinning antifungal effects.

In *C. heterostrophus*, exposure to isothiocyanates led to a distinct proteomic profile characterized by the downregulation of virulence-related pathways, including energy metabolism, melanin biosynthesis, and cell wall integrity. Mutants lacking oxidative stress regulators, such as ChTRX2, exhibited heightened sensitivity, suggesting a key role for redox homeostasis in fungal adaptation to these compounds. This integration of proteomics and genetics highlights key mechanisms of fungal adaptation to stress and suggests potential targets for enhancing antifungal strategies [5].

Comparisons of proteomic profiles between the conidial and mycelial stages of *Trichophyton rubrum* have further expanded our understanding of fungal responses to environmental and chemical stressors. This dermatophyte, responsible for common superficial infections, exhibits distinct proteomic profiles across its life stages. High-resolution mass spectrometry identified over 4000 proteins, revealing significant stage-specific differences. Aerobic metabolism and protein synthesis pathways were most active in the mycelial stage, while secretory proteases were abundant in conidia, reflecting their roles in infection and survival. Bioinformatic analyses, including KEGG pathway mapping, elucidated how acetylation patterns influence these adaptations and highlighted potential targets for antifungal therapies [38].

Advances in proteomic and bioinformatics methodologies have significantly deepened our knowledge of fungal responses to biocontrol agents (Table 1). Bioinformatics plays a critical role in the processing and interpretation of large proteomic datasets, enabling the identification of differentially expressed proteins and their associated pathways. Techniques such as KEGG pathway mapping and protein–protein interaction network analysis have unraveled how fungi adapt to antifungal compounds and modify their metabolism and cellular processes in response to stress [5,38]. These studies reveal the intricate adaptations that fungi employ to neutralize antifungal compounds and suggest novel targets for sustainable disease management strategies. By combining proteomics with other omics approaches, researchers can further elucidate the molecular underpinnings of these interactions, creating new opportunities for sustainable agricultural practices.

## 6. The Role of Proteomics in Sustainable Agriculture: Insights from Recent Studies

**Figure 4:**
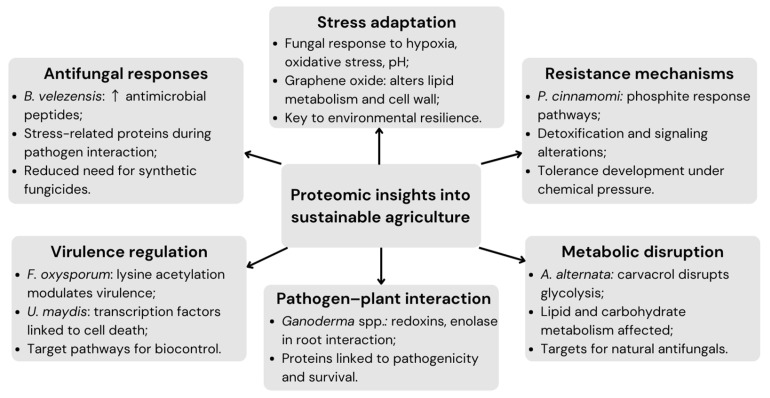
Proteomic insights supporting sustainable agricultural practices.

Proteomics has emerged as a powerful tool in modern agriculture, particularly for sustainable practices aimed at reducing dependency on chemical inputs [4,34]. By examining how crops, pathogens, and biocontrol agents interact at the molecular level, proteomics can offer significant insights into enhancing agricultural productivity and sustainability [5,20,39], as illustrated in Figure 4, which summarizes the main proteomic findings reported in recent studies related to antifungal responses, stress adaptation, resistance mechanisms, and pathogen–host interactions.

In recent years, proteomic approaches have been widely utilized to explore biocontrol mechanisms, pathogen resistance, and stress tolerance in crops. For instance, the antifungal properties of *Bacillus velezensis* strains have been characterized, revealing that lipopeptides produced by these bacteria enhance their antagonistic activity by increasing proteins associated with antimicrobial compound biosynthesis and stress responses during pathogen interaction [7]. This demonstrates the potential of microbial biocontrol agents to reduce reliance on chemical fungicides, which contribute to resistance and environmental harm, thus promoting more sustainable farming practices. However, challenges such as low stability and environmental degradation limit the effectiveness of lipopeptides in field applications. In this context, nanotechnology emerges as a promising strategy, as it can increase the stability of lipopeptides, control their release, and improve their antimicrobial efficiency, as demonstrated in studies on the association of antimicrobial peptides with nanoparticles [40].

Proteomics also plays a key role in identifying fungal responses to environmental stresses, which are essential for the development of sustainable agricultural practices. The reconstruction of the gene regulatory network of *U. maydis* revealed transcription factors involved in fungal cell death processes, providing a basis for targeting key regulatory pathways to control fungal infections in crops [41]. In this approach, subtractive proteomics was employed to identify glutamate–cysteine ligase (GCL) as a novel antifungal target in *Taphrina deformans*, the causative agent of peach leaf curl disease [29]. These studies discovered unique metabolic pathways essential for fungal survival and pathogenicity, guiding the design of targeted fungicides like polyoxin D and strobilurins.

Such insights support the development of environmentally sustainable management strategies for fungal diseases in agriculture. Moreover, a study on the hypoxic adaptation of *Paracoccidioides lutzii* demonstrated how hypoxia-related proteins regulate fungal survival under oxygen-limited conditions, a relevant factor in agricultural soils [42]. Following this direction, the proteomic study of surface proteins of *B. cinerea* identified those involved in pathogenicity, offering potential new targets for eco-friendly fungicide development [4]. The identification of key fungal proteins linked to virulence and resistance provides a foundation for developing targeted and environmentally sustainable strategies for disease management [43,44].

*F. oxysporum*, a pathogen responsible for wilt diseases in many crops, has been the subject of proteomic and transcriptomic investigations. Proteomic analysis can reveal key insights into pathogen metabolic pathways and the plant’s response to stress, thus guiding the design of resistance strategies that reduce the need for pesticides [34]. Reducing pesticide use is essential for more sustainable agricultural practices, as excessive pesticide application leads to environmental contamination, human health risks, and the development of resistance to pests and pathogens. Sustainable agricultural strategies based on proteomic insights can help mitigate these issues by increasing plant resistance and promoting environmentally friendly disease management techniques.

In line with these findings, the identification of the critical role of lysine acetylation in the virulence of *F. oxysporum* provides valuable insights into potential molecular targets for broad-spectrum fungicides [32]. Similarly, proteomic analyses of phytopathogenic fungi have elucidated key resistance mechanisms and potential targets for biocontrol strategies. For instance, the proteomic response of *Phytophthora cinnamomi* to phosphite, a widely used fungicide, revealed metabolic and signaling pathway alterations associated with phosphite resistance [5]. Likewise, *Ganoderma boninense* and *Ganoderma tornatum* exhibited differential expression of proteins such as enolase and redoxins during interactions with oil palm roots, linking these proteins to pathogenicity and fungal survival [25]. Furthermore, the impact of carvacrol on *A. alternata* demonstrated that this natural compound disrupts glycolysis and carbohydrate metabolism, which are essential processes for fungal growth. These findings highlight the role of proteomics in identifying metabolic vulnerabilities for biocontrol, reducing dependence on chemical pesticides, and promoting environmentally sustainable disease management [20].

By integrating traditional and modern agricultural practices, proteomics facilitates the development of targeted antifungal strategies that reduce reliance on broad-spectrum fungicides while mitigating their environmental impact and enhancing crop productivity. Proteomic analyses have revealed fungal adaptations to environmental stressors such as oxidative stress, temperature fluctuations, pH variations, and nutrient limitations, providing valuable insights into fungal resilience mechanisms [44]. These findings support the development of eco-friendly antifungals by targeting key metabolic pathways or stress-responsive proteins. For instance, graphene oxide has demonstrated potent antifungal activity against *F. graminearum* by disrupting lipid metabolism, impairing cell wall synthesis, and altering carbon and nitrogen flows, highlighting its potential as a sustainable alternative to conventional fungicides [45]. Additionally, exploring mitochondrial regulatory elements, such as self-splicing introns in pathogenic fungi, may offer novel molecular targets for antifungal intervention [44].

## 7. Concluding Remarks

Proteomics has been employed as a tool for studying pathogenic fungi, providing valuable information on their responses to antifungal agents, environmental stresses, and interactions with host plants. The application of proteomics in agriculture provides molecular-level insights into fungal pathogenicity, resistance mechanisms, and stress adaptation, contributing to the evaluation of both chemical and biological control agents. By enabling the identification of key proteins and pathways involved in these processes, proteomics supports the development of more targeted and eco-friendly antifungal strategies. These insights are crucial for advancing sustainable agriculture, improving crop protection, and promoting a healthier environment.

## Figures and Tables

**Table 1:** Overview of proteomic studies on biocontrol agents and their antifungal mechanisms *.

Biocontrol Agent	Target Fugus	Proteomic Technique	Main Proteins/Pathways	Key Findings	Reference
Nunamycin and Nunapeptin (nonribosomal peptides)	*Rhizoctonia solani*, *Pythium aphanidermatum*	MALDI-TOF imaging mass spectrometry	Cellular pathways, vital processes	Nunamycin and nunapeptin disrupt fungal cellular processes; peptide synthesis is environmentally influenced	[33]
Benzothiazole (microbial volatile)	*Botrytis cinerea*	iTRAQ	Mitochondrial membrane, glyoxylate cycle, ATP generation	Benzothiazole undermines mitochondrial function and energy homeostasis, affecting key fungal processes	[6]
VOCs from *Saccharomyces cerevisiae* CR-1	*Phyllosticta citricarpa*	2D-PAGE and LC-MS/MS	Glycolysis, TCA cycle, oxidative stress response	VOCs impair energy metabolism and oxidative stress defenses, highlighting critical vulnerabilities	[11]
VOCs from *Bacillus subtilis* CF-3	*Colletotrichum gloeosporioides*	TMT-based quantitative proteomics	Cell wall integrity, energy metabolism, ergosterol biosynthesis	VOCs disrupted metabolic pathways and ergosterol biosynthesis, inhibiting fungal growth	[8]
Fengyncins from *Bacillus subtilis* BS155	*Magnaporthe grisea*	Comparative proteomics	Mitochondrial membrane potential, ROS production, chromatin condensation	Fengycins induced ROS accumulation, chromatin condensation, and mitochondrial dysfunction, causing cell death	[36]
Peptides from a defensin protein (DefSm2-D) expressed in *Silybum marianum*	*Fusarium graminearum*	ESI-MS, circular dichroism spectroscopy	Membrane permeabilization, cell wall integrity	Defensins disrupted fungal membranes and induced morphological changes in fungal cell walls	[19]
Lipopeptides (surfactin, bacillomycin, fengycin)	*Diaporthe* spp.	Nano-LC-MS/MS	Stress-related proteins, lipid metabolism, amino acid transport	Lipopeptides induce stress responses and lipid remodeling, affecting fungal physiology and resistance	[7]
Lipopeptides (surfactin, bacillomycin, fengycin)	*Fusarium oxysporum*	iTRAQ	Mitochondrial membrane, energy metabolism pathways	Lipopeptides disrupt mitochondrial processes and energy metabolism, emphasizing antifungal potential	[37]
Antifungal protein PgAFP	*Penicillium expansum*	Label-free quantitative proteomics	Oxidative stress, patulin biosynthesis	PgAFP induced oxidative stress and increased patulin production, raising caution for its application	[30]

* Abbreviations: MALDI-TOF, matrix-assisted laser desorption/ionization time-of-flight; iTRAQ, isobaric tags for relative and absolute quantitation; TMT, tandem mass tag; 2D-PAGE, bidimensional polyacrylamide gel electrophoresis; ESI, electrospray ionization; VOCs, volatile organic compounds.

## Data Availability

No new data were created or analyzed in this study. Data sharing is not applicable to this article.
